# Stochastic Model for Phonemes Uncovers an Author-Dependency of Their Usage

**DOI:** 10.1371/journal.pone.0152561

**Published:** 2016-04-08

**Authors:** Weibing Deng, Armen E. Allahverdyan

**Affiliations:** 1 Complexity Science Center & Institute of Particle Physics, Central China Normal University, Wuhan 430079, China; 2 Yerevan Physics Institute, Alikhanian Brothers Street 2, Yerevan 375036, Armenia; University of Illinois-Chicago, UNITED STATES

## Abstract

We study rank-frequency relations for phonemes, the minimal units that still relate to linguistic meaning. We show that these relations can be described by the Dirichlet distribution, a direct analogue of the ideal-gas model in statistical mechanics. This description allows us to demonstrate that the rank-frequency relations for phonemes of a text do depend on its author. The author-dependency effect is not caused by the author’s vocabulary (common words used in different texts), and is confirmed by several alternative means. This suggests that it can be directly related to phonemes. These features contrast to rank-frequency relations for words, which are both author and text independent and are governed by the Zipf’s law.

## Introduction

Language can be viewed as a hierarchic construction: phoneme, syllable, morpheme, word … Each of these objects expresses meaning or participates in its formation, and consists of elements of the previous level, i.e. syllable consists of phonemes [1–3].

The lowest hierarchic level is phoneme, which is defined to be a representative for a group of sounds that are not distinguishable with respect to their meaning-formation function in a concrete language. For instance */r/* and */l/* are different phonemes in English, e.g. because *row* and *low* which differ only by these phonemes are different words; see S1 Appendix for a list of English phonemes. But they are the same phoneme in Japanese, since in that language there is no danger of meaning-ambiguity upon mixing */r/* with */l/*. (Different speech sounds that are realizations of the same phoneme are known as allophones.) Thus the meaning is crucial for the definition of the phoneme, although a single phoneme does not express a separate meaning [1–3]. The next hierarchic level (syllable) indirectly participates in the definition of the phoneme, since the syllable bounds phonemes, i.e. there cannot be a phoneme which belongs to two different syllables; e.g. diphthongs belong to the same syllable [1, 2].

The history of phoneme is a rich and complex one. It appeared in Greek and Indian linguistic traditions simultaneously with atomistic ideas in natural philosophy [4–6]. Analogies between atom and phoneme are still potent in describing complex systems [7, 8]. Within the Western linguistic tradition the development of phoneme was for a while overshadowed by related (but different) concepts of letter and sound [1, 2]. The modern definition of phoneme goes back to late XIX century [2]. While it is agreed that the phoneme is a unit of linguistic analysis [3], its psychological status is a convoluted issue [9–13]. Different schools of phonology and psychology argue differently about it, and there is a spectrum of opinions concerning the issue (e.g. perception of phonemes, their identification, reproduction *etc*) [12, 13]; see [9–11] for recent reviews.

For defining a rank-frequency relation, one calculates the frequencies *f*_*r*_ of certain constituents (e.g. words or phonemes) entering into a given text, lists them in a decreasing order

$$\begin{array}{c} f_{1}\geq f_{2}\geq ...\geq f_{n}, \end{array}$$ (1)

and studies the dependence of the frequency *f*_*r*_ on the rank *r* (its position in Eq (1), 1 ≤ *r* ≤ *n*). This provides a coarse-grained description, because not the frequencies of specific phonemes are described, but rather the order relation between them, e.g. the same form of the rank-frequency relation in two different texts is consistent with the same phoneme having different frequencies in those texts. The main point of employing rank-frequency relations is that they (in contrast to the full set of frequencies) can be described via simple statistical models with very few parameters.

Rank-frequency relations are well-known for words, where they comply to the Zipf’s law; see [14, 15] for reviews. This law is universal in the sense that for all sufficiently long texts (and their mixtures, i.e. corpora) it predicts the same power law shape *f*_*r*_ ∝ *r*^−1^ for the dependence of the word frequency on its rank. It was shown recently that the representation of the word frequencies via hidden frequencies—the same idea as employed in the present work—is capable of reproducing both the Zipf’s law and its generalizations to low-frequency words (hapax legomena) [16]. Due to its universality, the Zipf’s law for words cannot relate the text to its author.

The rank-frequency relation for morphemes and syllables was so far not studied systematically. Ref. [17] comes close to this potentially interesting problem, since it studies the rank-frequency relations of Chinese characters, which are known to represent both morpheme and syllable (in this context see also [18–20]). This study demonstrated that the Zipf’s law still holds for a restricted range of ranks. For long texts this range is relatively small, but the frequencies in this range are important, since they carry out ≃ 40% of the overall text frequency. It was argued that the characters in this range refer to the most polysemic morphemes [17].

There are also several works devoted to the rank-frequency relations of phonemes and letters [21–27]. One of first works is that by Sigurd, who has shown that the phoneme rank-frequency relations are not described by the Zipf’s law [21]. He also noted that a geometric distribution gives a better fit than the Zipf’s law. Other works studied various few-parameter functions—e.g. the Yule’s distribution—and fitted it to the rank-frequency relations for phonemes of various languages; see [27] for a recent review of that activity.

The present work has two motivations. First, we want to provide an accurate description of rank-frequency relation for phonemes. It is shown that such a description is provided by postulating that phoneme frequencies are random variables with a given density. The ranked frequencies are then recovered via the order statistics of this density. This postulate allows to restrict the freedom of choosing various (theoretical) forms of rank-frequency relations, since—as developed in mathematical statistics [28, 29]—the idea of the simplest density for *probability of probability* allows to come up with the unique family of Dirichlet densities. This family is characterized by a positive parameter *β*, which allows quantitative comparison between phoneme frequencies for different authors. From the physical side, the Dirichlet density is a direct analogue of the ideal gas model from statistical mechanics, while *β* relates to the inverse temperature. Recall that the ideal-gas model provides a simple and fruitful description of the coarse-grained (thermodynamic) features of matter starting from the principles of atomic and molecular physics [30]. Thus we substantiate the atom-phoneme metaphor, that so far was developed only qualitatively [7, 8].

Our second motivation for studying rank-frequency relations for phonemes is whether they can provide information on the author of the text, and thereby attempt at clarifying the psychological aspect of phonemes. As seen below, the Dirichlet density not only leads to an accurate description of phoneme rank-frequency relations, but it also allows to establish that the frequencies of phonemes do depend on the author of the text. We corroborate this result by an alternative means.

The closest to the present approach is the study by Good [22] which was developed in Refs. [23–25]. These authors applied the same idea on hidden probabilities as here, but they restricted themselves by the flat density, which is a particular case *β* = 1 of the Dirichlet density [22–24]. Superficially, this case seems to be special, because it incorporates the idea of non-informative (unknown) probabilities (in the Bayesian sense) [31]. However, the development of the Bayesian statistics has shown that the *β* = 1 case of the Dirichlet density is by no means special with respect the prior information [31]. Rather, the whole family of Dirichlet densities (with *β* > 0 being a free parameter) qualifies for this role [32].

This paper is organized as follows. Next section discusses the Dirichlet density and its features. There we also deduce explicit formulas for the probabilities ordered according to the Dirichlet density. Then, we analyze the data obtained from English texts written by different authors and show that it can be described via the Dirichlet density. There we also demonstrate (in different ways including non-parametric methods) that rank-frequency relations for phonemes are author-dependent. Next, we show that the author-dependency effect is not caused by common words used in different texts. We summarize in the last section.

## Materials and Methods

### Ideal-gas models

The general idea of applying ideal-gas models in physics [30] is that for describing coarse-grained features of certain physical systems, interactions between their constituents (atoms or molecules) can be accounted for superficially (in particular, neglected to a large extent). Instead, one focusses on the simplest statistical description that contains only a few parameters (e.g. temperature, volume, number of particles etc) [30]. In physics this simplest descriptions amounts to the Gibbs density [30]. Its analogue in mathematical statistics is known as the Dirichlet density, and is explained below. Ideal gas models in physics are useful not only for gases—where interactions are literally weak—but also for solids, where interactions are important, but their detailed structure is not, and hence it can be accounted for in a simplified way [30].

Following these lines, we apply below the Dirichlet density to phoneme frequencies observed in a given text. More precisely, the ordered frequencies generated by the Dirichlet density are compared with observed (and ordered) frequencies of phonemes in a given text. This ordering of frequencies amounts to a rough and simplified account of (inter-phoneme) interactions, and suffices for an accurate description of the rank-frequency relations for phonemes; see below.

### Definition and main features of Dirichlet density

The Dirichlet density $D(\theta_{1},...,\theta_{n})$ is a probability density over continuous variables (*θ*_1_, …, *θ*_*n*_) which by themselves have the meaning of probabilities, i.e. $D(\theta_{1},...,\theta_{n})$ is non-zero only for *θ*_*k*_ ≥ 0 and $\sum_{k=1}^{n}\theta_{k}=1$:

$$\begin{array}{c} D\left(\theta_{1},...,\theta_{n}|\beta_{1},...,\beta_{n}\right)=\frac{\Gamma [\sum_{k=1}^{n}\beta_{k}]}{\prod_{k=1}^{n}\Gamma \left[\beta_{k}\right]}\prod_{k=1}^{n}\theta_{k}^{\beta_{k}-1}\;\delta \left(\sum_{k=1}^{n}\theta_{k}-1\right), \end{array}$$ (2)

where *β*_*k*_ > 0 are the parameters of the Dirichlet density, *δ*(*x*) is the delta-function, $\Gamma \left[x\right]=\int_{0}^{\infty }d\theta \;\theta^{x-1}e^{-\theta }$ is the Euler’s Γ-function, and Eq (2) is properly normalized: $\int_{0}^{\infty }\prod_{k=1}^{n}d\theta_{k}D\left(\theta_{1},...,\theta_{n}|\beta_{1},...,\beta_{n}\right)=1$.

The random variables Θ_1_, …, Θ_*n*_ (with realizations *θ*_1_, …, *θ*_*n*_) are independent modulo the constraint that they sum to 1; see Eq (2). In this sense Eq (2) is the simplest density for probabilities. Now Eq (2) for a particular case *β*_*k*_ = *β* (which is most relevant for our purposes) can be given the following statistical-physics interpretation: if $\ln(\frac{1}{\theta_{k}})$ is interpreted as the energy of *k* [33–35], then *β* − 1 becomes the inverse temperature for an ideal gas. It is useful to keep this analogy in mind, when discussing further features of the Dirichlet density.

Consider the subset (*θ*_1_, …, *θ*_*m*_) (*m* < *n*) of probabilities (*θ*_1_, …, *θ*_*n*_). If (*θ*_1_, …, *θ*_*m*_) should serve as new probabilities, they should be properly normalized. Hence we define new random variables as follows:

$$\begin{array}{c} \left({\tilde{\theta }}_{1},...,{\tilde{\theta }}_{n}\right)=\left({\hat{\theta }}_{1},...,{\hat{\theta }}_{m},\theta_{m+1},...\theta_{n}\right),\;\;\;{\hat{\theta }}_{k}=\frac{\theta_{k}}{\sum_{i=1}^{m}\theta_{i}},\;\;k=1,...,m. \end{array}$$ (3)

The joint probability $P({\tilde{\theta }}_{1},...,{\tilde{\theta }}_{n})$ now reads from Eq (2):

$$\begin{array}{c} P\left({\tilde{\theta }}_{1},...,{\tilde{\theta }}_{n}\right)=D\left({\hat{\theta }}_{1},...,{\hat{\theta }}_{m}|\beta_{1},...,\beta_{m}\right)\;X\left(\theta_{m+1},...,\theta_{n}\right), \end{array}$$ (4)

where the precise form of X is not relevant for the message of Eq (4): if we disregard some probabilities and properly re-normalize the remaining ones, the kept probabilities follow the same Dirichlet density and are independent from the disregarded ones [28]. This means that we do not need to know the number of constituents before applying the Dirichlet density. This feature is relevant for phonemes, because their exact number is to a large extent a matter of convention, e.g. should English diphthongs be regarded as separate phonemes, or as combinations of a vowel and a semi-vowel.

Condition Eq (4) (called sometimes neutrality), together with few smoothness conditions, determines the shape Eq (2) of the Dirichlet density [29].

Assuming *n* free parameters *β*_*k*_ for *n* phoneme frequencies does not amount to any effective description. Hence below we employ Eq (2) with

$$\begin{array}{c} \beta_{k}=\beta , \end{array}$$ (5)

for describing the ranked phoneme frequencies. This implies that the full vector (*β*_1_, …, *β*_*n*_) is replaced by a certain characteristic value *β*, which is to be determined from comparing with data. To provide some intuition on *β*, let us note from Eq (2) that a larger value of *β* leads to more homogeneous density (many events have approximately equal probabilities). For *β*_*k*_ → 0 the region *θ*_*k*_ ≃ 0 is the most probable one.

### Distribution of ordered probabilities (order statistics)

The random variables Θ_1_, …, Θ_*n*_ (whose realizations are *θ*_1_, …, *θ*_*n*_ in Eq (2)) are now put in a non-increasing order:

$$\begin{array}{c} \Theta_{\left(1\right)}\geq ...\geq \Theta_{\left(n\right)}. \end{array}$$ (6)

This procedure defines new random variables, so called order statistics of the original ones [36]. We are interested by the marginal probability density of Θ_(*r*)_. It is difficult to obtain this object explicitly, because the initial Θ_1_, …, Θ_*n*_ are correlated random variables. However, we can explicitly obtain from Eq (2) a two-argument function that suffices for calculating the moments of Θ_(*r*)_ [see S2 Appendix]:

$$\begin{array}{c} \chi_{r}\left(y;m\right)=\frac{\Gamma [n\beta ]}{\Gamma [n\beta +m]}\;\frac{n!}{(n-r)!(r-1)!}\;\frac{y^{\beta -1}e^{-y}}{\Gamma [\beta ]}\;\varphi^{n-r}\left(y\right){\left[1-\varphi (y)\right]}^{r-1}, \end{array}$$ (7)

where Γ[*x*] is the Γ-function and where

$$\begin{array}{c} \varphi \left(y\right)=\frac{1}{\Gamma [\beta ]}\int_{0}^{y}dx\;x^{\beta -1}e^{-x} \end{array}$$ (8)

is the regularized incomplete Γ-function. Now the moments of Θ_(*r*)_ are obtained as

$$\begin{array}{c} \langle \theta_{\left(r\right)}^{m}\rangle =\int_{0}^{\infty }dy\;y^{m}\chi_{r}\left(y;m\right). \end{array}$$ (9)

**Fig 1:**
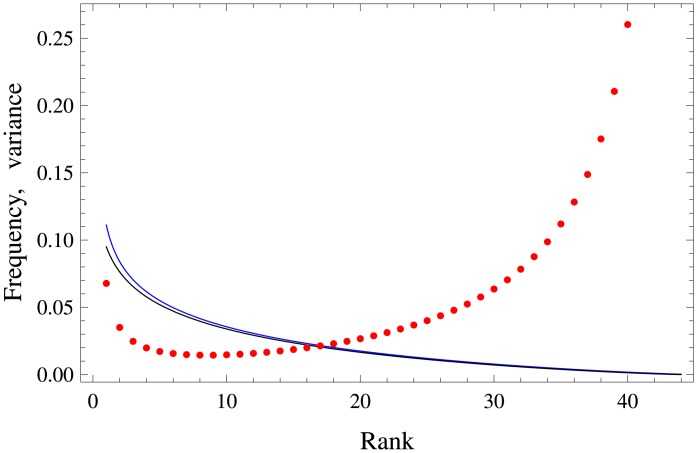
Rank-frequency curves and error generated by the Dirichlet density with *β* = 0.8 and *n* = 44. Blue curve: 〈*θ*_(*r*)_〉 (as a function of *r*) calculated according to Eqs (7)–(9). Black curve: ${\hat{f}}_{r}$ calculated via the approximate formula Eq (10); cf. S2 Appendix. Red points: the normalized variance $\left(\langle \theta_{\left(r\right)}^{2}\rangle -{\langle \theta_{\left(r\right)}\rangle }^{2}\right)/{\langle \theta_{\left(r\right)}\rangle }^{2}$ for *r* = 1, …, 44 calculated according to Eqs (7)–(9). This expression is well approximated in S2 Appendix.

In the next section we shall see that the sequence of ordered probabilities *f*_*r*_ [cf. Eq (1)] can be generated via Eq (7). To this end, the empiric quantities *f*_*r*_ will be compared to ${\hat{f}}_{r}=\langle \theta_{\left(r\right)}\rangle$; cf. Eq (9). The rationale for using the average is that for parameters we are interested in—where *n* ≃ 40 − 50 (for English phonemes *n* = 44) and 0.5 ≤ *β* ≤ 1—we get from Eqs (7)–(9) that relative fluctuations around the average ${\hat{f}}_{r}\equiv \langle \theta_{\left(r\right)}\rangle$ are small. Namely, $\varepsilon_{r}\equiv \left(\langle \theta_{\left(r\right)}^{2}\rangle -{\langle \theta_{\left(r\right)}\rangle }^{2}\right)/{\langle \theta_{\left(r\right)}\rangle }^{2}≲0.02$ for all values of *r*, excluding *r* ≈ *n*, i.e. very low frequency phonemes. This is shown in Fig 1 for a particular value *β* = 0.8. Note that *ε*_*r*_ is not a monotonic function of *r*: it is smallest for middle ranks. (Even for those values of *r*, where *ε*_*r*_ ≃ 1, ${\hat{f}}_{r}=\langle \theta_{\left(r\right)}\rangle$ can still describe the empiric frequencies *f*_*r*_, as seen below.) Now there is a simpler approximate formula for ${\hat{f}}_{r}=\langle \theta_{\left(r\right)}\rangle$ that is deduced from Eq (9) [see S2 Appendix]:

$$\begin{array}{c} \frac{r}{n}=1-\varphi \left({\hat{f}}_{r}n\beta \right). \end{array}$$ (10)

Fig 1 shows that ${\hat{f}}_{r}$ obtained from Eq (10) indeed approximates well 〈*θ*_(*r*)_〉 for almost all ranks *r*.

## Results and Discussions

### Fitting rank-frequency relations to the Dirichlet distribution

We studied 48 English texts written by 16 different, native-English authors; see Table 1 and S3 Appendix. For each text we extracted the phoneme frequencies ${\left\{f_{r}\right\}}_{r=1}^{n}$ and ordered them as in Eq (1); the list of English phonemes is given in S1 Appendix. The transcription of words into phonemes was carried out via the software PhoTransEdit, which is available at [37]. This is a relatively slow, but very robust software, since it works by checking each word in the phonetic dictionary. Thus it can err only on those unlikely cases, when the word is not found in the dictionary.

**Table 1:** Nine texts and their parameters. Texts are abbreviated and numbered. *N*_*tw*_, *N*_*pht*_, *N*_*dw*_ and *N*_*phd*_ are, respectively, the total number of words, the number of phonemes of the total words, the number of different words and the number of phonemes of different words. J. Austen: *Mansfield Park* (MP or 1) 1814; *Pride and Prejudice* (PP or 2) 1813; *Sense and Sensibility* (SS or 3) 1811. C. Dickens: *A Tail of Two Cities* (TC or 4) 1859; *Great Expectations* (GE or 5) 1861; *Adventures of Oliver Twist* (OT or 6) 1838. J. Tolkien: *The Fellowship of the Ring* (FR or 7) 1954; *The Return of the King* (RK or 8) 1955; *The Two Towers* (TT or 9) 1954.

Texts	*N*_*tw*_	*N*_*pht*_	*N*_*dw*_	*N*_*phd*_
MP (1)	160473	567750	7854	48747
PP (2)	121763	435322	6385	39767
SS (3)	119394	425822	6264	38668
TC (4)	135420	468642	9841	58760
GE (5)	186683	623079	10933	65364
OT (6)	159103	555372	10359	61072
FR (7)	177227	617106	8644	46509
TT (8)	143436	502303	7676	39823
RK (9)	134462	431141	7087	36494

It is important to specify from which set of words (of a text) one extracts the phoneme frequencies. Two natural choices are possible here: either one employs all words of the text, or different words of the text (i.e. multiple occurrences of the same word are neglected). We shall study both cases. For clarity reasons, we shall present our results by focussing on the three authors mentioned in Table 1; see also Tables 2 and 3 in this context. Three texts by three authors is in a sense the minimal set-up for described effects. We stress that other texts we studied fully corroborate our results; they are partially described in Table 4 below and in S3 Appendix.

**Table 2:** Fitting parameters for texts numbered as 1–9; see Eqs (11) and (12) and Table 1 for text numbers. The phoneme frequencies are extracted from all words of the text.

Parameters	1	2	3	4	5	6	7	8	9
*β*	0.61	0.63	0.61	0.67	0.69	0.69	0.75	0.74	0.79
*SS*_*err*_ × 10^7^	7696	7574	6151	4317	5287	3993	4196	4337	3580
*R*^2^	0.9768	0.9765	0.9816	0.9859	0.9820	0.9867	0.9844	0.9842	0.9860

**Table 3:** Fitting parameters for texts numbered as 1–9; see Eqs (11) and (12) and Table 1 for text numbers. The phoneme frequencies are extracted from different words of the text; see Table 2 for the values of *β* calculated from all words of texts. Eqs (17) and (18) compare the data presented in Tables 2 and 3.

Parameters	1	2	3	4	5	6	7	8	9
*β*	0.72	0.69	0.69	0.77	0.78	0.79	0.968	0.979	0.975
*SS*_*err*_ × 10^7^	5150	4495	5003	6107	5265	5220	11296	12943	10366
*R*^2^	0.9818	0.9847	0.9829	0.9771	0.9800	0.9800	0.9501	0.9403	0.9525

**Table 4:** The values of *β* extracted from different words of texts for 5 authors. For each author we analyzed three texts. They are described in the S3 Appendix, where we also discuss 8 other authors.

Author	*β*		
C. Lyell	0.798	0.785	0.792
A. R. Wallace	0.744	0.756	0.739
C. Darwin	0.817	0.810	0.822
H. Spenser	0.646	0.658	0.650
H. G. Wells	0.737	0.735	0.724

The ordered set ${\left\{f_{r}\right\}}_{r=1}^{n}$ of phoneme frequencies for each text was compared with the prediction ${\left\{{\hat{f}}_{r}=\langle \theta_{\left(r\right)}\rangle \right\}}_{r=1}^{n}$ of the Dirichlet density [see Eq (9)]. Here the parameter *β* [cf. Eqs (2) and (5)] is found from minimizing the error:

$$\begin{array}{c} SS_{err}=\sum_{k=1}^{n}{\left(f_{k}-{\hat{f}}_{k}\right)}^{2}. \end{array}$$ (11)

For each studied case we also monitored the coefficient of correlation between ${\left\{f_{r}\right\}}_{r=1}^{n}$ and ${\left\{{\hat{f}}_{r}\right\}}_{r=1}^{n}$:

$$\begin{array}{c} R^{2}=\frac{{\left[\;\sum_{k=1}^{n}\left(f_{k}-\bar{f}\right)\left({\hat{f}}_{k}-\bar{\hat{f}}\right)\;\right]}^{2}}{\sum_{k=1}^{n}{\left(f_{k}-\bar{f}\right)}^{2}\sum_{k=1}^{n}{\left({\hat{f}}_{k}-\bar{\hat{f}}\right)}^{2}}, \end{array}$$ (12)

where

$$\begin{array}{c} \bar{f}\equiv \frac{1}{n}\sum_{k=1}^{n}f_{k},\;\bar{\hat{f}}\equiv \frac{1}{n}\sum_{k=1}^{n}{\hat{f}}_{k}. \end{array}$$ (13)

A good fitting means that *R*^2^ is close to 1. We found that (as functions of *β*) *SS*_*err*_ and 1 − *R*^2^ minimize simultaneously.

**Fig 2:**
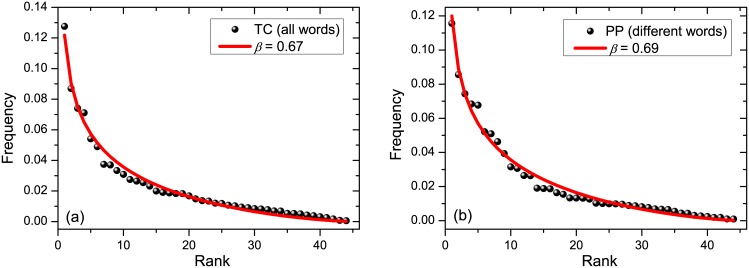
Rank-frequency relation (black circles) and the fitting with Dirichlet distribution (red line). (a) Left figure: text TC, where frequencies were extracted from all words. (b) Right figure: text PP, where different words were employed; see Table 1 for the description of texts.

Examples of fitting curves for phoneme rank-frequency relations are presented in Fig 2. The fitting parameters are given in Tables 2 and 3. Note that the fitting values of *R*^2^ are good. The group of most frequent eight phonemes reads [see S1 Appendix]: /ı/, /ə/, /n/, /s/, /t/, /l/, /d/, /r/. The concrete ranking between them depends on the text, but the most frequent one is normally /ı/.

**Fig 3:**
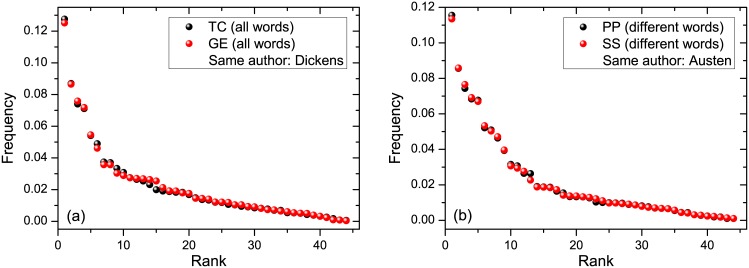
Rank-frequency relation (black and red circles) for two texts written by the same author. (a) Left figure: TC and GE written by Dickens (all words were employed for extracting the phoneme frequencies). (b) Right figure: PP and SS written by Austen (different words were employed); see Table 1.

**Fig 4:**
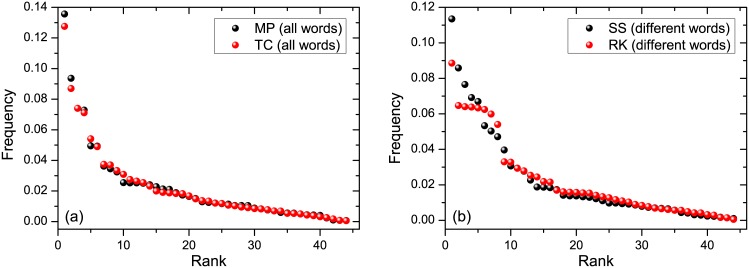
Rank-frequency relation (black and red circles) for two texts written by different authors. (a) Left figure: TC by Dickens *versus* MP by Austen (all words were employed). (b) Right figure: SS by Austen *versus* RK by Tolkien (different words were employed); see Table 1 for parameters of these texts.

Tables 2 and 3 show that the texts by the same author have closer values of *β* than those written by different authors; see also Figs 3 and 4. This can be quantified via the following three inequalities

$$\begin{array}{ccc} & & 0<b\left(A\right)\equiv \text{min}\;\{|\beta_{i}-\beta_{k}{\left|\right\}}_{i=1,2,3;k=4,5,6,7,8,9}\;-\;\text{max}\;\{|\beta_{i}-\beta_{j}{\left|\right\}}_{i<j;i,j=1,2,3}, \end{array}$$ (14)

$$\begin{array}{ccc} & & 0<b\left(D\right)\equiv \text{min}\;\{|\beta_{i}-\beta_{k}{\left|\right\}}_{i=4,5,6;k=1,2,3,7,8,9}\;-\;\text{max}\;\{|\beta_{i}-\beta_{j}{\left|\right\}}_{i<j;i,j=4,5,6}, \end{array}$$ (15)

$$\begin{array}{ccc} & & 0<b\left(T\right)\equiv \text{min}\;\{|\beta_{i}-\beta_{k}{\left|\right\}}_{i=7,8,9;k=1,2,3,4,5,6}\;-\;\text{max}\;\{|\beta_{i}-\beta_{j}{\left|\right\}}_{i<j;i,j=7,8,9}, \end{array}$$ (16)

where A, D and T refer, respectively to Austen, Dickens and Tolkien [see Table 1]. The indices *i* and *j* run over the texts by the same author, while *k* refer to different authors, e.g. *i*, *j* = {1, 2, 3} (Austen) and *k* = {4, 5, 6, 7, 8, 9} (not Austen). The minimization (or maximization) in Eqs (14)–(16) goes over indicated indices.

Eqs (14)–(16) hold both for phoneme frequencies extracted from different words and from all words of a text; cf. Tables 2 and 3. For instance, *b*^[all words]^(A) = 0.02, *b*^[all words]^(D) = 0.02, *b*^[all words]^(T) = 0. The latter is the only minor exclusion from Eqs (14)–(16).

Thus the set ${\left\{\beta_{i}\right\}}_{i=1}^{9}$ fragments into three clusters that refer to different authors. Note that

$$\begin{array}{c} b^{\left[\text{diff}.\;\text{words}\right]}\left(a\right)>b^{\left[\text{all}\;\text{words}\right]}\left(a\right),\;a=A,\;D,\;T. \end{array}$$ (17)

Hence different words display the author-dependency in a stronger form; this is confirmed below by other methods.

The author-dependency of phoneme rank-frequency relation is unexpected, because the rank-frequency relation for words (which consists of phonemes) follows the Zipf’s law whose shape is independent of the author [14–16]. Note that the few most frequent phonemes and the least frequent ones appear to fit best the theoretical prediction; cf. Fig 2. This feature again contrasts the rank-frequency relation for words, where it is known that high-frequency words—these are mostly the functional words, e.g. *and*, *or*—do hold the Zipf’s law worst than other words [16]. On the other hand, the moderate-frequency phonemes deviate most from the prediction of the Dirichlet curve; cf. Fig 2. This effect is not statistical, since fluctuations around the average are most suppressed for moderate-frequency phonemes; see after Eq (9) and Figs 1 and 2.

Another pertinent result is that [see Tables 2 and 3]

$$\begin{array}{c} \beta_{i}^{\left[\text{diff}.\;\text{words}\right]}>\beta_{i}^{\left[\text{all}\;\text{words}\right]},\;\;i=1,...,9, \end{array}$$ (18)

i.e. the phoneme distribution obtained from different words is more homogeneous [see our discussion after Eq (5)], because for all words the frequency of high-rank phonemes is amplified due to multiple usage of frequent words.

Note that the above three texts belong to one genre (novels) and concern only three authors. Hence we studied other 13 native-English authors who created in XIX’th and the first half of XX’th century; see S3 Appendix. These additional studies corroborate the obtained results. In particular, Table 4 presents the values of *β* extracted from different texts of 5 authors. These authors were selected so that their language differences due to social, temporal and professional backgrounds are minimized. In addition, we selected 4 of them to be professional scientists, since the language of scientific works is normally more unified. Lyell, Darwin, Wallace, and Spenser were naturalists, while the fifth author (H.G. Wells) held a PhD in biology and wrote a lot about scientists. Lyell strongly influenced Darwin, while Darwin and Wallace were close colleagues. All these three naturalists influenced Spenser and Wells. However, Table 4 shows that the values of *β* for these 5 authors are clearly different and hold analogues of Eqs (14)–(16).

We want to stress that *β* anyhow changes in a bounded interval: 0.5 < *β* < 1. Hence if one takes sufficiently many authors, their values of *β* will start to overlap. In our study of (overall) 16 authors we confirmed this expectation; see S2 Appendix. However, these overlaps are accidental, i.e. the overlapping authors can be easily distinguished by alternative means. In particular, their phoneme distributions can be robustly distinguished via distances, as described below.

### Distance between phoneme frequencies

The author-dependency of phoneme rank-frequency relation is corroborated by looking directly at suitable distances between the ranked phoneme frequencies in different texts. We choose to work with the variational distance

$$\begin{array}{c} \rho_{1}\left(ij\right)=\frac{1}{2}\sum_{k=1}^{n}\left|\;f_{k}\left[i\right]-f_{k}\left[j\right]\;\right|, \end{array}$$ (19)

where ${\left\{f_{k}\left[i\right]\right\}}_{k=1}^{n}$ are the ordered phoneme frequencies in the text *i*. We shall also employ a more fine-grained (detail-specific) distance. Let *f*[*α*|*i*] be the frequency of phoneme *α* in text *i* (*α* = 1, …, *n*, *i* = 1, …, 9). We can now define [cf. Eq (19)]

$$\begin{array}{c} \rho_{0}\left(ij\right)=\frac{1}{2}\sum_{\alpha =1}^{n}\left|\;f\left[\alpha |i\right]-f\left[\alpha |j\right]\;\right|. \end{array}$$ (20)

Now *ρ*_0_(*ij*) = 0 only if *f*[*α*|*i*] = *f*[*α*|*j*]. It is seen from Tables 5–7 that *ρ*_0_(*ij*) > *ρ*_1_(*ij*), as it should be, because *ρ*_1_(*ij*) is less sensitive to details (i.e. it is more coarse-grained).

**Table 5:** Distances *ρ*_0_ and *ρ*_1_ between texts; see Table 1 and Eqs (20) and (19) for the definition of *ρ*_0_ and *ρ*_1_. The phoneme frequencies are extracted from all words of the text. Eqs (24) and (25) compare the distances from all words with those from different words.

Texts	*ρ*_0_ × 10^5^	*ρ*_1_ × 10^5^	Texts	*ρ*_0_ × 10^5^	*ρ*_1_ × 10^5^	Texts	*ρ*_0_ × 10^5^	*ρ*_1_ × 10^5^	Texts	*ρ*_0_ × 10^5^	*ρ*_1_ × 10^5^
1 & 2	3045	2227	1 & 4	3583	2784	2 & 7	7653	4978	4 & 7	5174	3950
1 & 3	2062	1602	1 & 5	4690	3044	2 & 8	7629	5052	4 & 8	5327	3568
2 & 3	2549	2103	1 & 6	4000	3260	2 & 9	7650	5449	4 & 9	5061	3935
4 & 5	3423	2100	1 & 7	7372	5149	3 & 4	3562	2546	5 & 7	6113	3894
4 & 6	2382	1978	1 & 8	7402	5227	3 & 5	4924	3022	5 & 8	6436	4014
5 & 6	3448	2753	1 & 9	7322	5599	3 & 6	4358	3181	5 & 9	6217	4325
7 & 8	2584	1808	2 & 4	3645	2712	3 & 7	7737	5266	6 & 7	5074	3727
7 & 9	2066	1809	2 & 5	4762	3059	3 & 8	6950	5085	6 & 8	5706	3934
8 & 9	2464	2037	2 & 6	4064	3110	3 & 9	7447	5654	6 & 9	5202	3770

**Table 6:** Distances *ρ*_0_ and *ρ*_1_ between texts; see Table 1 and Eqs (20) and (19). The phoneme frequencies are extracted from different words of the text; see Eqs (24) and (25) for comparison with all words.

Texts	*ρ*_0_ × 10^5^	*ρ*_1_ × 10^5^	Texts	*ρ*_0_ × 10^5^	*ρ*_1_ × 10^5^	Texts	*ρ*_0_ × 10^5^	*ρ*_1_ × 10^5^	Texts	*ρ*_0_ × 10^5^	*ρ*_1_ × 10^5^
1 & 2	1563	1346	1 & 4	2296	1967	2 & 7	8141	6587	4 & 7	5918	4795
1 & 3	1317	1205	1 & 5	2703	2110	2 & 8	9999	7544	4 & 8	7875	5971
2 & 3	1413	1346	1 & 6	2868	2470	2 & 9	9167	7136	4 & 9	6899	5368
4 & 5	1568	1266	1 & 7	7430	6103	3 & 4	2718	2193	5 & 7	5521	4631
4 & 6	1380	1126	1 & 8	9535	7200	3 & 5	3264	2486	5 & 8	7842	5566
5 & 6	1100	1052	1 & 9	8434	6775	3 & 6	3257	2636	5 & 9	6646	5222
7 & 8	2853	1653	2 & 4	2839	2252	3 & 7	7943	6539	6 & 7	5595	4486
7 & 9	1946	1476	2 & 5	3318	2436	3 & 8	9998	7447	6 & 8	7785	5645
8 & 9	2025	1569	2 & 6	3458	2709	3 & 9	8997	7022	6 & 9	6786	5201

**Table 7:** Distances *ρ*_0_ and *ρ*_1_ between texts; see Table 1 and Eqs (20) and (19). The phoneme frequencies are extracted from different words of each text after excluding the words that are common for both compared texts; see Eqs (26) and (27) for comparison with the situation without excluding common words.

Texts	*ρ*_0_ × 10^5^	*ρ*_1_ × 10^5^	Texts	*ρ*_0_ × 10^5^	*ρ*_1_ × 10^5^	Texts	*ρ*_0_ × 10^5^	*ρ*_1_ × 10^5^	Texts	*ρ*_0_ × 10^5^	*ρ*_1_ × 10^5^
1 & 2	3792	2832	1 & 4	4758	3912	2 & 7	13323	9469	4 & 7	10980	7025
1 & 3	3217	2463	1 & 5	5742	4276	2 & 8	15733	10387	4 & 8	13905	7371
2 & 3	3734	2502	1 & 6	6087	4830	2 & 9	14113	9621	4 & 9	12109	6928
4 & 5	3146	2190	1 & 7	12574	8800	3 & 4	5188	4344	5 & 7	10346	6537
4 & 6	2930	2215	1 & 8	15119	9576	3 & 5	5887	4917	5 & 8	13003	7021
5 & 6	2329	1610	1 & 9	13490	8895	3 & 6	6476	5285	5 & 9	11673	6673
7 & 8	5918	3317	2 & 4	5708	4529	3 & 7	13391	9835	6 & 7	10413	6580
7 & 9	4421	2773	2 & 5	6385	4991	3 & 8	15842	10637	6 & 8	13288	6667
8 & 9	4770	2809	2 & 6	6880	5495	3 & 9	14244	9891	6 & 9	11911	6433

To motivate the choice of the variational distance $\rho_{0}=\frac{1}{2}\sum_{\alpha =1}^{n}\left|\;p_{\alpha }-q_{\alpha }\;\right|$ between two sets of probabilities ${\left\{p_{\alpha }\right\}}_{\alpha =1}^{n}$ and ${\left\{q_{\alpha }\right\}}_{\alpha =1}^{n}$, let us recall an important feature of this distance [38]: $\rho_{0}={\text{max}}_{\Omega }|\sum_{\alpha \in \Omega }\left(p_{\alpha }-q_{\alpha }\right)|$, where the maximization goes over all sub-sets *Ω* of {1, …, *n*}. Thus *ρ*_0_ refers to the (composite) event that gives the largest probability difference between ${\left\{p_{\alpha }\right\}}_{\alpha =1}^{n}$ and ${\left\{q_{\alpha }\right\}}_{\alpha =1}^{n}$.

Tables 5 and 6 refer, respectively, to phoneme frequencies extracted from all words and different words of the text. These tables show that phoneme rank-frequency relations between the texts written by the same author are closer to each other—in the sense of distances *ρ*_0_ and *ρ*_1_—than the ones written by different authors. This is also seen on Figs 3 and 4.

To quantify these differences, consider the following inequalities that define clustering with respect to authors (see Table 1 for numbering of texts, and note that *ρ*_λ_(*ij*) = *ρ*_λ_(*ji*) for the distance between the texts *i* and *j*):

$$\begin{array}{ccc} & & 0<z_{\lambda }\left(A\right)\equiv \text{min}\;{\left\{\rho_{\lambda }\left(ik\right)\right\}}_{i=1,2,3;k=4,5,6,7,8,9}\;-\;\text{max}\;{\left\{\rho_{\lambda }\left(ij\right)\right\}}_{i<j;i,j=1,2,3},\;\;\lambda =0,1, \end{array}$$ (21)

$$\begin{array}{ccc} & & 0<z_{\lambda }\left(D\right)\equiv \text{min}\;{\left\{\rho_{\lambda }\left(ik\right)\right\}}_{i=4,5,6;k=1,2,3,7,8,9}\;-\;\text{max}\;{\left\{\rho_{\lambda }\left(ij\right)\right\}}_{i<j;i,j=4,5,6},\;\;\lambda =0,1, \end{array}$$ (22)

$$\begin{array}{ccc} & & 0<z_{\lambda }\left(T\right)\equiv \text{min}\;{\left\{\rho_{\lambda }\left(ik\right)\right\}}_{i=7,8,9;k=1,2,3,4,5,6}\;-\;\text{max}\;{\left\{\rho_{\lambda }\left(ij\right)\right\}}_{i<j;i,j=7,8,9},\;\;\lambda =0,1, \end{array}$$ (23)

where A, D and T refer, respectively to Austen, Dickens and Tolkien; cf. Eqs (21)–(23) with Eqs (14)–(16). For example, the maximal distance Eq (20) between texts by Austen (see Table 1) is denoted by $\text{max}\;{\left\{\rho_{0}\left(ij\right)\right\}}_{i<j;i,j=1,2,3}$, while $\text{min}\;{\left\{\rho_{0}\left(kl\right)\right\}}_{k=1,2,3;l=4,5,6,7,8,9}$ is the minimal distance between texts written by Austen and those written by Dickens and Tolkien. Note that Eqs (21)–(23) hold as well for other 13 authors we analyzed; see S3 Appendix for examples.

The meaning of Eqs (21)–(23) can be clarified by looking at an authorship attribution task: let several texts *i* = 1, 2, 3 by (for example) Austen are at hands, and one is given an unknown text *α*. The question is whether *α* could also be written by Austen. If now max_*i*_ [*ρ*_λ_(*iα*)] ≤ max_*i*<j_ [*ρ*_λ_(*ij*)], we have an evidence that *α* is written by Austen.

We stress that there are no fitting parameters in Eqs (20)–(23). Our data (cf. Tables 5 and 6) holds eleven (out of twelve) inequalities Eqs (21)–(23) for phoneme frequencies extracted both from different and from all words of the text. There is only one exclusion: $z_{1}^{\left[\text{all}\;\text{words}\right]}\left(T\right)=-0.00207$, which is by an order of magnitude smaller than the respective frequencies [cf. Eq (23)]. Apart of this minor exclusion, we confirm the above prediction (obtained via the fitted values of *β*) on the author-dependency for phoneme frequencies.

Data shown in Tables 5 (all words) and 6 (different words) also imply the following inequalities [confirming Eq (17)]

$$\begin{array}{c} z_{\lambda }^{\left[\text{diff}.\;\text{words}\right]}\left(a\right)>z_{\lambda }^{\left[\text{all}\;\text{words}\right]}\left(a\right),\;\lambda =0,1,\;a=A,\;D,\;T. \end{array}$$ (24)

Another pertinent feature is that the distances *ρ*_0_ and *ρ*_1_ between texts written by the same author hold

$$\begin{array}{ccc} & & \rho_{\lambda }^{\left[\text{all}\;\text{words}\right]}\left(ij\right)>\rho_{\lambda }^{\left[\text{diff}.\;\text{words}\right]}\left(ij\right),\\ & & \lambda =0,1,\;(ij)=\{\;(12),(13),(23),(45),(46),(78),(79),(89)\;\}. \end{array}$$ (25)

Seventeen out of eighteen relations Eq (25) hold for our data; see Tables 5 and 6, where we present the distances *ρ*_0_ and *ρ*_1_ for phoneme frequencies deduced from, respectively, all words and different words of the texts. The only exclusion in Eq (25) is $\rho_{0}^{\left[\text{diff}.\;\text{words}\right]}\left(78\right)-\rho_{0}^{\left[\text{all}\;\text{words}\right]}\left(78\right)=0.02853-0.02584=0.00269$. No definite relations exist between $\rho_{\lambda }^{\left[\text{all}\;\text{words}\right]}$ and $\rho_{\lambda }^{\left[\text{diff}.\;\text{words}\right]}$ for texts written by different authors. One can interpret Eq (25) as follows. When going from different words to all words of the text, the majority of frequent words are not author-specific: they are mostly key-words (that are specific to the text, but not necessarily to the author) and functional words (e.g. *and*, *or*, *of*, *but*) that are again not author-specific.

Taken together, Eq (24) and Eq (25) imply that the clustering with respect to authors is better visible for frequencies extracted from different words of the texts (the inter-cluster distance increases, whereas the intra-cluster distance decreases). The same effect was obtained above via fitted values of *β*’s; see Eq (17).

### The origin of the author-dependency effect is not in common words

One possible reason for the author-dependency of phoneme frequencies is that the effect is due to the vocabulary of the author. In this scenario the similarity between phoneme frequencies in text written by the same author would be caused by the fact that these texts have sufficiently many common words that carry out the same phonemes.

Texts written by the same author do have a sizeable number of common words, as was already noted within the authorship attribution research [39, 40]. We confirm this result in Table 8, where it is seen that the fraction of common words holds the analogues of Eqs (21)–(23). Hence this fraction also shows the author-dependency effect.

**Table 8:** The fraction *p* of common words between texts given in Table 1. Now *p* is defined as follows. Let *n*(*i*) and *n*(*ij*) be, respectively, the number of different words in text *i* and the number of common words in texts *i* and *j*. We define: *p*(*ij*) = *n*(*ij*)/(*n*(*i*) + *n*(*j*) − *n*(*ij*)), where 0 ≤ *p*(*ij*) ≤ 1. This is the number of common words divided over the number of all different words in texts *i* and *j*. As seen from the data below, analogues of Eqs (21)–(23) hold with 1 − *p*(*ij*) instead of *ρ*_λ_(*ij*).

Texts	*p* × 10^5^	Texts	*p* × 10^5^	Texts	*p* × 10^5^	Texts	*p* × 10^5^
1 & 2	47554	1 & 4	35592	2 & 7	26549	4 & 7	33901
1 & 3	47786	1 & 5	35819	2 & 8	24180	4 & 8	30387
2 & 3	50655	1 & 6	36660	2 & 9	24643	4 & 9	32005
4 & 5	41146	1 & 7	28978	3 & 4	33463	5 & 7	32069
4 & 6	42454	1 & 8	25870	3 & 5	32813	5 & 8	27963
5 & 6	41822	1 & 9	26730	3 & 6	34643	5 & 9	29994
7 & 8	45010	2 & 4	32902	3 & 7	27572	6 & 7	32002
7 & 9	46948	2 & 5	32499	3 & 8	25340	6 & 8	28649
8 & 9	48173	2 & 6	33877	3 & 9	25733	6 & 9	30518

In order to understand whether the author-dependency of phoneme frequencies can be explained via common words, we excluded from different words of texts *i* and *k* the common words of those texts [*i*, *k* = 1, …, 9, see Table 1], re-calculated phoneme frequencies, and only then determined the respective distances $\rho_{0}^{\left[\text{no}\;\text{comm}.\;\text{words}\right]}\left(ik\right)$ and $\rho_{1}^{\left[\text{no}\;\text{comm}.\;\text{words}\right]}\left(ik\right)$. If the explanation via common words holds, they will not show author-dependency. This is however not the case: the effect is there because relations Eqs (21)–(23) do hold for them

$$\begin{array}{c} z_{\lambda }^{\left[\text{no}\;\text{comm}.\;\text{words}\right]}\left(a\right)>0,\;\lambda =0,1,\;a=A,\;D,\;T. \end{array}$$ (26)

Eq (26) is deduced from Table 7, where we present the distances *ρ*_0_ and *ρ*_1_ for the situation, where the common words are excluded.

After excluding the common words the author-dependency did not get stronger in the sense of Eq (25), because the data of Tables 6 (different words) and 7 (excluded common words) imply for texts written by the same author

$$\begin{array}{ccc} & & \rho_{\lambda }^{\left[\text{no}\;\text{comm}.\;\text{words}\right]}\left(ij\right)>\rho_{\lambda }^{\left[\text{diff}.\;\text{words}\right]}\left(ij\right),\;\lambda =0,1,\\ & & \;(ij)=\{\;(12),(13),(23),(45),(46),(78),(79),(89)\;\}. \end{array}$$ (27)

In this context recall Eqs (24) and (25). But it also did not get weaker [cf. Eq (24) and Eqs (21)–(23)], because

$$\begin{array}{c} z_{\lambda }^{\left[\text{no}\;\text{comm}.\;\text{words}\right]}\left(a\right)>z_{\lambda }^{\left[\text{diff}.\;\text{words}\right]}\left(a\right),\;\lambda =0,1,\;a=A,\;D,\;T, \end{array}$$ (28)

as seen from Tables 6 and 7, which refer, respectively, to different words and the excluded common words.

## Conclusion

Phonemes are the minimal building blocks of the linguistic hierarchy that still relate to meaning. A coarse-grained description of phoneme frequencies is provided by rank-frequency relations. For describing these relations we followed the qualitative analogy between atoms and phonemes [7, 8]. Atoms amount to a finite (and not very large) number of discrete elements from which the multitude of substances and materials are built [30]. Likewise, a finite number of phonemes can construct a huge number of texts [8].

The simplest description of an (sufficiently dilute) atomic system is provided via the ideal gas model [30]. By studying 16 native-English authors, we show that the rank-frequency relations for phonemes can be described via the ordered statistics of the Dirichlet density, the direct analogue of the ideal gas model in statistics. In particular, though the number of phonemes is not very large (English has 44 phonemes), it is just large enough to validate the statistical description. The single parameter of the Dirichlet density corresponds to the (inverse) temperature of the ideal gas in statistical physics. It appears that the most frequent phonemes fit the Dirichlet distribution much better than others. This contrasts to the rank-frequency relations for words, where the Zipf’s law holds worst for the most frequent words.

The fitting to the Dirichlet density uncovers an important aspect of phoneme frequencies: they depend on the author of the text. This fact is seen for authors who created their works in various genres (novels, scientific texts, journal papers), and also for authors whose language-dependence on social, temporal and educational background has been minimized (e.g. the closely inter-related group of English naturalists including Darwin, Wallace, Lyell, and Spencer). We confirmed this result via a parameter-free method that is based on calculating distances between phoneme frequencies of different texts. Again, this contrasts to the Zipf’s law for rank-frequency relations of words whose shape is author-independent.

It is well-known that certain aspects of text-statistics display author-dependency, and this is applied in various author attribution tasks; see e.g. [38–43] for recent reviews. In particular, this concerns frequencies of functional words. The fact that author-dependency is seen on such a coarse-grained level as rank-frequency relations may mean that phoneme frequencies can be useful for existing methods of authorship attribution [40–43]. This should be clarified in future.

A straightforward reason for explaining the author-dependency effect of phoneme frequencies would be that it is due to the author’s vocabulary, as reflected by common words in texts written by the same author. The previous section has shown that such an explanation is ruled out.

Then we are left with options that the effect is due to storing (with different frequencies) syllables or/and phonemes. If syllable frequencies have author-dependency, this could result to author-dependent phoneme frequencies, because there are specific rules that (at least probabilistically) determine the phoneme composition of syllables [44]. But note that syllables are in several respect similar to words (and not phonemes): *(i)* there are many of them; e.g. English has more than 12000 syllables. *(ii)* There is large gap between frequent and infrequent syllables [45, 46] (cf. with the hapax legomena for words). *(iii)* There are indications that syllables are stored in a syllabic lexicon that in several ways is similar to the mental lexicon that stores words [45, 46].

The second possibility would mean that the authors store phonemes [11], and this will provide a statistical argument for psychological reality of phonemes. Note that the issue of psychological reality of a phoneme is not settled in modern phonology and psychology, various schools arguing pro and contra of it; see [9–13] for discussions. And then both these options might be present together. Thus further research—also involving rank-frequency relations for syllables—is needed for clarifying the situation.

The presented methods can find applications in animal communication systems. In this context, we recall an interesting argument [47]. The number of phonemes in languages roughly varies between 20 and 50. Indeed, the average number of phonemes in European languages is ≃ 37. (English has 44 phonemes, but if diphthongs are regarded as combinations of a vowel and a semi-vowel this number reduces to 36.) In tonal languages the overall number of phonemes is larger, e.g. it is ∼180 for Chinese. (The tone produces phonemes and not allophones, since they do change the meaning.) But the number of phonemes without tones still complies with the above rough bound. Since Old Chinese (spoken in 11 to 7’th centuries B.C.) lacked tones, the tonal phonemes of modern Chinese evolved from their non-tonal analogues that complies with the above number [48]. By its order of magnitude this number (∼20 − 50) coincides [47] with the number of ritualized (i.e. sufficiently abstract) signals of animal communication, which is also stable across different species [49]. (An example of this are gestures of apes.) This number is sufficiently large to invite the application of the presented statistical methods to signals of animal communication. And the stability of this number may mean that there are further similarities (to be yet uncovered) between phonemes and ritualized signals.

## Supporting Information

S1 Appendix A list of English phonemes. (PDF)

S2 Appendix Order statistics for Dirichlet density. (PDF)

S3 Appendix Information on the other 13 authors and 39 texts. (PDF)
