# Supplementary material for: Stochastic Model for Phonemes Uncovers an Author-Dependency of Their Usage
Source: PLoS One. 2016 Apr 8;11(4):e0152561. doi: 10.1371/journal.pone.0152561 (PMC4825982; doi:10.1371/journal.pone.0152561)
Supplement: S1 Appendix — (PDF) [file pone.0152561.s001.pdf]

S1 Appendix. A list of English phonemes

Here we recall 44 English phonemes according to the International Phonetic Alphabet.

I. 20 vowels (7 short phonemes, 5 long and 8 diphtongs):

ʌ (but), æ (cat), ə (about), e (men), ɪ (sit), ɒ (not), ʊ (book),

ɑ: (part), ɜ: (word, learn), i: (read), ɔ: (sort), u: (too),

aɪ (my), aʊ (how), oʊ (go), eɪ (day), ɪə (here), oɪ (boy), ʊə (tour, pure), eə (wear, fair)

II. 24 consonants:

b (born), d (do), f (five), g (get), h (house), j (yes), k (cat), l (lion), m (mouse), n (nouse), ɳ (sing), p (put), r (room), s (saw), ʃ (shall), t (time), ʧ (church), θ (think), ð (the), v (very), w (window), z (zoo), ʒ (casual), dʒ (judge)
