# Supplementary material for: Stochastic Model for Phonemes Uncovers an Author-Dependency of Their Usage
Source: PLoS One. 2016 Apr 8;11(4):e0152561. doi: 10.1371/journal.pone.0152561 (PMC4825982; doi:10.1371/journal.pone.0152561)
Supplement: S2 Appendix — (PDF) [file pone.0152561.s002.pdf]

S2 Appendix. Order statistics for Dirichlet density

Let us introduce the following notation for the order integration

$$\mathcal{I}(\mathrm{d}\theta_1, ..., \mathrm{d}\theta_n) \equiv \int_0^\infty \mathrm{d}\theta_1 \int_0^{\theta_1} \mathrm{d}\theta_2 ... \int_0^{\theta_{n-1}} \mathrm{d}\theta_n. \tag{1}$$

Now the average over the order statistics of the Dirichlet density is defined as

$$\langle \theta_{(r)}^m \rangle = \frac{\mathcal{I}(\mathrm{d}\theta_1, ..., \mathrm{d}\theta_n)\, \theta_r^m \,\delta(\sum_{k=1}^n \theta_k - 1) \prod_{k=1}^n \theta_k^{\beta-1}}{\mathcal{I}(\mathrm{d}\theta_1, ..., \mathrm{d}\theta_n)\, \delta(\sum_{k=1}^n \theta_k - 1) \prod_{k=1}^n \theta_k^{\beta-1}}. \tag{2}$$

In the numerator of (2) we change variables as $\hat{\theta}_k = r\theta_k$ $(r > 0)$, multiply both sides by e^{-r}, and then integrate both sides over $r \in [0, \infty)$:

$$\mathcal{I}(\mathrm{d}\theta_1, ..., \mathrm{d}\theta_n)\, \theta_r^m \,\delta(\sum_{k=1}^n \theta_k - 1) \prod_{k=1}^n \theta_k^{\beta-1} \times \Gamma[n\beta + m] = \mathcal{I}(\mathrm{d}\hat{\theta}_1, ..., \mathrm{d}\hat{\theta}_n)\, \hat{\theta}_r^m \prod_{k=1}^n \hat{\theta}_k^{\beta-1} e^{-\hat{\theta}_k}. \tag{3}$$

The denominator of (2) is worked out analogously.

Let us now define

$$\chi_r(y; m) = \frac{\Gamma[n\beta]}{\Gamma[n\beta + m]} \frac{\mathcal{I}(\mathrm{d}\hat{\theta}_1, ..., \mathrm{d}\hat{\theta}_n)\, \delta(y - \hat{\theta}_r) \prod_{k=1}^n \hat{\theta}_k^{\beta-1} e^{-\hat{\theta}_k}}{\mathcal{I}(\mathrm{d}\hat{\theta}_1, ..., \mathrm{d}\hat{\theta}_n) \prod_{k=1}^n \hat{\theta}_k^{\beta-1} e^{-\hat{\theta}_k}} \tag{4}$$

so that the following relation holds

$$\langle \theta_{(r)}^m \rangle = \int_0^\infty \mathrm{d}y\, y^m \chi_r(y; m). \tag{5}$$

This is the equation (9) of the main text. Working out $\mathcal{I}(\mathrm{d}\hat{\theta}_1, ..., \mathrm{d}\hat{\theta}_n) \prod_{k=1}^n \hat{\theta}_k^{\beta-1} e^{-\hat{\theta}_k}$ and $\mathcal{I}(\mathrm{d}\hat{\theta}_1, ..., \mathrm{d}\hat{\theta}_n)\, \delta(y - \hat{\theta}_r) \prod_{k=1}^n \hat{\theta}_k^{\beta-1} e^{-\hat{\theta}_k}$ in (4) via integration by parts (starting from the last integration in $\mathcal{I}(\mathrm{d}\hat{\theta}_1, ..., \mathrm{d}\hat{\theta}_n)$) we obtain equations (7–9) of the main text.

If $(n-r) \gg 1$ and $r \gg 1$ the behavior of $\chi_r(y; m)$ in equations (7) of the main text is determined by the exponential factor $e^{(n-r)\ln\varphi(y) + r\ln(1-\varphi(y))}$. Working it out via the saddle-point method we conclude that asymptotically:

$$\chi_r(y; m) \simeq \frac{\Gamma[n\beta]}{\Gamma[n\beta + m]} \frac{1}{\sqrt{2\pi\sigma}} e^{-\frac{1}{2\sigma}(y-y_0)^2}, \tag{6}$$

where y_0 and σ are defined as follows

$$\frac{n-r}{n} = \varphi(y_0), \qquad \sigma = \frac{(n-r)r}{n^3} \frac{1}{[\,\varphi'(y_0)\,]^2}, \tag{7}$$

where $\phi'(y) = \mathrm{d}\varphi(y)/\mathrm{d}y$.

Hence we get from (5) and (6, 7):

$$\langle \theta_{(r)} \rangle = \frac{y_0}{n\beta}, \tag{8}$$

$$\langle \theta_{(r)}^2 \rangle - \langle \theta_{(r)} \rangle^2 = \frac{n\beta\sigma - y_0^2}{[n\beta]^2(n\beta + 1)} = \frac{1}{[n\beta]^2(n\beta + 1)} \left(\frac{\beta(n-r)r}{n^2} \frac{1}{[\varphi'(y_0)]^2} - y_0^2 \right). \tag{9}$$

The importance of fluctuations is characterized by

$$\frac{\langle\theta^2_{(r)}\rangle - \langle\theta_{(r)}\rangle^2}{\langle\theta_{(r)}\rangle^2} = \frac{1}{n\beta+1}\left(\frac{\beta(n-r)r}{n^2}\,\frac{1}{y_0^2[\varphi'(y_0)]^2} - 1\right) \tag{10}$$

$$= \frac{1}{n\beta+1}\left(\frac{\beta(n-r)r}{n^2}\,\Gamma^2[\beta]\,y_0^{-2\beta}\,e^{2y_0} - 1\right), \tag{11}$$

where we employed

$$\varphi(y) = \frac{1}{\Gamma[\beta]}\int_0^y \mathrm{d}x\, x^{\beta-1}e^{-x} \tag{12}$$

This is the equation (8) of the main text. Eq. (11) is a good approximation of $\frac{\langle\theta^2_{(r)}\rangle - \langle\theta_{(r)}\rangle^2}{\langle\theta_{(r)}\rangle^2}$ calculated (exactly) from equations (7-9) of the main text; see Fig. 1 of the main text.
