# Supplementary material for: Stochastic Model for Phonemes Uncovers an Author-Dependency of Their Usage
Source: PLoS One. 2016 Apr 8;11(4):e0152561. doi: 10.1371/journal.pone.0152561 (PMC4825982; doi:10.1371/journal.pone.0152561)
Supplement: S3 Appendix — (PDF) [file pone.0152561.s003.pdf]

S3 Appendix. Information on the other 13 authors and 39 texts

Here are the works by the other 13 English writers we studied in addition to the authors described in Table 1 of the main text. After the title of each work we give its writing/publication date, the number of different words, and the number of phonemes of different words. The table below summarizes the values of β for phonemes extracted from different words of each text.

Charlotte Bronte: *Jane Eyre* (1847, 12488, 75933), *Shirley* (1849, 14481, 88911), *Villette* (1853, 14176, 88025).

Clive S. Lewis: *Perelandra* (1943, 7030, 41265), *Out of the Silent Planet* (1938, 6045, 35371), *That Hideous Strength* (1946, 7842, 46618).

George Eliot: *Adam Bede* (1859, 9685, 56819), *Romola* (1862, 13402, 83255), *The Mill on the Floss* (1860, 11682, 72071).

George MacDonald: *Paul Faber, Surgeon* (1879, 9615, 57634), *There and Back* (1891, 8807, 51865), *Unspoken Sermons, Series I-III* (1867–1889), 7815, 47674).

Alfred R. Wallace: *Contributions to the Theory of Natural Selection* (1870, 6829, 43619), *Man's Place in the Universe* (1904, 5626, 35738), *The Malay Archipelago* (1869, 8785, 52760).

Charles Darwin: *On the Origin of Species* (1859, 6764, 42519), *The Descent of Man, and Selection in Relation to Sex* (1871, 13069, 82027), *The Voyage of the Beagle* (1839, 11359, 69667).

Herbert G. Wells: *Marriage* (1912, 12076, 76705), *The Country of the Blind, and Other Stories* (1894-1909, 11537, 71091), *The New Machiavelli* (1911, 12702, 81773).

Herbert Spenser: *The principle of psychology* (1855, 6932, 48036), *The Principles of Ethics* (1897, 10575, 71729), *The Principles of Sociology* (1874, 15215, 98353).

Joseph R. Kipling: *A Diversity of Creatures* (1912, 9993, 57358), *From Sea to Sea; Letters of Travel* (1889, 15165, 91038), *Indian Tales* (1890, 11975, 69231).

Oscar Wilde: *A Critic in Pall Mall Being Extracted from Reviews and Miscellanies* (1919, 8168, 49299), *Miscellanies* (1908, 8204, 50539), *The Picture of Dorian Gray* (1891, 6725, 37933).

Charles Lyell: *A Manual of Elementary Geology* (1852, 9573, 61983), *The Antiquity of Man* (1863, 9280, 59868), *The Student's Elements of Geology* (1865, 10347, 67742).

Walter Scott: *Ivanhoe, A Romance* (1819, 11857, 71974), *Old Mortality* (1816, 12049, 73894), *Rob Roy* (1817, 12524, 76175).

William M. Thackeray: *The History of Pendennis* (1848, 15591, 96039). *The Virginians* (1857, 15158, 92548). *Vanity Fair* (1848, 14695, 90373).

The table shows that the values of β for several authors do overlap. These overlaps are accidental, as can be verified by calculating the distances. Here are some examples for authors whose values of β overlap:

$$\max{}_{\text{Darwin}}[\rho_0] = 0.001155, \qquad \max{}_{\text{Thackeray}}[\rho_0] = 0.0097, \tag{13}$$

$$\min{}_{\text{Darwin vs. Thackeray}}[\rho_0] = 0.01508, \tag{14}$$

where $\max_{\text{Darwin}}[\rho_0]$ is the maximal ρ_0-distance between the 3 texts by Darwin [see (20) of the main text for the definition of ρ_0], $\max_{\text{Thackeray}}[\rho_0]$ is the same quantity for the texts by Thackeray, and $\min_{\text{Darwin vs. Thackeray}}[\rho_0]$ is the minimal ρ_0-distance between the texts of Darwin versus those of Thackeray. It is seen that although the values of β for Darwin and Thackeray overlap, the distances between phoneme frequencies do cluster, and they hold analogues of (21–23) of the main text, i.e. $\max_{\text{Darwin}}[\rho_0] < \min_{\text{Darwin vs. Thackeray}}[\rho_0]$ and $\max_{\text{Thackeray}}[\rho_0] < \min_{\text{Darwin vs. Thackeray}}[\rho_0]$.

TABLE I: The values of β extracted from different words of each text. The order of texts corresponds to the description, i.e. the three texts by C. Bronte in the table (from left to right) refer, respectively, to *Jane Eyre*, *Shirley*, and *Villette*.

Author	β		
C. Bronte	0.762	0.767	0.758
C. S. Lewis	0.781	0.780	0.778
G. Eliot	0.747	0.741	0.748
G. MacDonald	0.773	0.773	0.766
A. R. Wallace	0.744	0.756	0.739
C. Darwin	0.817	0.810	0.822
H. G. Wells	0.737	0.735	0.724
H. Spenser	0.646	0.658	0.650
J. R. Kipling	0.868	0.852	0.872
O. Wilde	0.793	0.785	0.803
C. Lyell	0.798	0.785	0.792
W. Scott	0.808	0.795	0.787
W. M. Thackeray	0.818	0.815	0.818

Similar relations hold for the ρ_1 distance [see (19) of the main text]:

$$\max_{\rm Darwin}[\rho_1] = 0.01674, \qquad \max_{\rm Thackeray}[\rho_1] = 0.00943, \tag{15}$$

$$\min_{\rm Darwin\, vs.\, Thackeray}[\rho_1] = 0.01705, \tag{16}$$

We give several other examples of distances for those authors whose values of β overlap. We found that all these examples hold the above clustering feature.

$$\max_{\rm Lyell}[\rho_0] = 0.03976, \qquad \max_{\rm MacDonald}[\rho_0] = 0.02557, \tag{17}$$

$$\min_{\rm Lyell\, vs.\, MacDonald}[\rho_0] = 0.04632, \tag{18}$$

$$\max_{\rm Lyell}[\rho_1] = 0.02442, \qquad \max_{\rm MacDonald}[\rho_1] = 0.01015, \tag{19}$$

$$\min_{\rm Lyell\, vs.\, MacDonald}[\rho_1] = 0.02968. \tag{20}$$

$$\max_{\rm Wallace}[\rho_0] = 0.02508, \qquad \max_{\rm Eliot}[\rho_0] = 0.02483, \tag{21}$$

$$\min_{\rm Wallace\, vs.\, Eliot}[\rho_0] = 0.02821, \tag{22}$$

$$\max_{\rm Wallace}[\rho_1] = 0.0135, \qquad \max_{\rm Eliot}[\rho_1] = 0.01108, \tag{23}$$

$$\min_{\rm Wallace\, vs.\, Eliot}[\rho_1] = 0.02047. \tag{24}$$

$$\max_{\rm Wilde}[\rho_0] = 0.0161, \qquad \max_{\rm Scott}[\rho_0] = 0.01801, \tag{25}$$

$$\min_{\rm Wilde\, vs.\, Scott}[\rho_0] = 0.02851, \tag{26}$$

$$\max_{\rm Wilde}[\rho_1] = 0.01901, \qquad \max_{\rm Scott}[\rho_1] = 0.01455, \tag{27}$$

$$\min_{\rm Wilde\, vs.\, Scott}[\rho_1] = 0.01954. \tag{28}$$
